# Relationship between Orthostatic Hypotension and White Matter Hyperintensity Load in Older Patients with Mild Dementia

**DOI:** 10.1371/journal.pone.0052196

**Published:** 2012-12-19

**Authors:** Hogne Soennesyn, Dennis W. Nilsen, Ketil Oppedal, Ole Jacob Greve, Mona K. Beyer, Dag Aarsland

**Affiliations:** 1 Centre for Age-Related Medicine, Stavanger University Hospital, Stavanger, Norway; 2 Department of Cardiology, Stavanger University Hospital, Stavanger, Norway; 3 Institute of Medicine, University of Bergen, Bergen, Norway; 4 Department of Electrical Engineering and Computer Science, University of Stavanger, Stavanger, Norway; 5 Department of Radiology, Stavanger University Hospital, Stavanger, Norway; 6 Department of Radiology and Nuclear Medicine, Oslo University Hospital, Oslo, Norway; 7 MoodNet Research Group, Stavanger University Hospital, Stavanger, Norway; 8 Department of Neurobiology, Ward Sciences and Society, Alzheimer’s Disease Research Centre, Karolinska Institute, Stockholm, Sweden; Cardiff University, United Kingdom

## Abstract

**Background/Objectives:**

White matter hyperintensities (WMH) in magnetic resonance imaging (MRI) scans of the brain, and orthostatic hypotension (OH) are both common in older people. We tested the hypothesis that OH is associated with WMH.

**Design:**

Cross-sectional study.

**Setting:**

Secondary care outpatient clinics in geriatric medicine and old age psychiatry in western Norway.

**Participants:**

160 older patients with mild dementia, diagnosed according to standardised criteria.

**Measurements:**

OH was diagnosed according to the consensus definition, measuring blood pressure (BP) in the supine position and within 3 minutes in the standing position. MRI scans were performed according to a common protocol at three centres, and the volumes of WMH were quantified using an automated method (n = 82), followed by manual editing. WMH were also quantified using the visual Scheltens scale (n = 139). Multiple logistic regression analyses were applied, with highest vs. lowest WMH quartile as response.

**Results:**

There were no significant correlations between WMH volumes and systolic or diastolic orthostatic BP drops, and no significant correlations between Scheltens scores of WMH and systolic or diastolic BP drops. In the multivariate analyses, only APOEε4 status remained a significant predictor for WMH using the automated method (p = 0.037, OR 0.075 (0.007–0.851)), whereas only age remained a significant predictor for WMH scores (p = 0.019, OR 1.119 (1.018–1.230)).

**Conclusion:**

We found no association between OH and WMH load in a sample of older patients with mild dementia.

## Introduction

White matter hyperintensities (WMH) are commonly found in cerebral T2-weighted magnetic resonance imaging (MRI) scans in older people [1], [2]. WMH seem to have a common distribution regardless of underlying diagnosis [3]–[4], with a preference for areas of lower relative perfusion. They have been associated with depression [5] and dementia [6]. WMH predict functional decline in voiding, mobility and cognition, and depression [7]–[9].

WMH have been associated, although only modestly [10], with classic cardiovascular risk factors [2], [11] including hypertension [12] and APOEε4 [13], and are considered a marker of cerebrovascular disease. Alternatively, WMH may, at least in Alzheimer’s disease (AD), primarily be associated with neurodegenerative disease [14]. However, some studies [15]–[19] suggest that hypotension, including orthostatic hypotension, plays a role in the development of WMH.

Orthostatic hypotension (OH) [20] is common in older people [21], and particularly in older people with dementia [22], [23]. OH is associated with falls [24], coronary heart disease and increased mortality [25].

Furthermore, one older study using CT scans found seated systolic blood pressure (BP) below 130 to be predictive of having white matter low attenuation (equivalent to WMH in MRI) of the brain [26], suggesting that the absolute BP level might be of importance.

In this study we wanted to explore the association between OH and WMH in older people with mild dementia. We hypothesized that systolic and/or diastolic BP drop at baseline are positively correlated with total WMH volumes and Scheltens deep WMH scores, and that having OH, or standing systolic BP at or below 110 mm Hg at baseline is independently associated with having more severe WMH on imaging. Since OH appears to be particularly common in Lewy body dementias [27], we tested this association separately.

## Methods

### Subjects

Consecutive referrals to dementia clinics in the counties of Rogaland and Hordaland in western Norway from March 2005 to March 2007 were screened, and patients with a first time diagnosis of mild dementia, i.e. a minimum Mini-Mental State Examination (MMSE) score of 20 were included. From April 2007 we selectively recruited patients with dementia with Lewy bodies (DLB) and Parkinson’s disease with dementia (PDD) fulfilling the aforementioned criteria of mild dementia. A total of 246 patients have completed baseline assessments, the last of whom was included in May 2011. In the current study, we included those who had both OH measurements and available MRI scans with adequate scan quality.

### Ethics Statement

The study was approved by the Regional Committee for Medical Research Ethics, Western Norway and the Norwegian authorities for collection of medical data. The subjects provided written consent to participate after the study procedures had been explained in detail to them and a caregiver, usually the spouse or offspring.

### Dementia Diagnosis

The diagnoses for AD, DLB, PDD and vascular dementia (VaD) were made according to consensus criteria [28]–[31], and for frontotemporal dementia (FTD) and alcoholic dementia according to the Lund-Manchester criteria [32] and the DSM-IV criteria, respectively. DLB and PDD were combined into one group (Lewy body dementia, LBD), because these conditions have several clinical and biological similarities [29], [33].

The diagnostic procedures and comprehensive standardised assessment have been described elsewhere [34]. Patients with acute delirium or terminal illness, as well as those recently diagnosed with a major somatic illness, previous bipolar disorder or psychotic disorder were excluded.

### Blood Pressure Measurements

Blood pressures were measured at baseline only, using an analogue sphygmomanometer. The protocol did not require a period of rest prior to the BP measurements. In the majority of patients, BP was measured once with the subject in the supine position, and then once (all patients) within 3 minutes after standing up. In some patients, the non-standing BP measurements were made in the sitting position (22/80 in the volumetry group, and 60/134 in the semi-quantitative group). For n = 9 patients the non-standing position is unknown.

Orthostatic hypotension (OH) was defined according to the consensus as a reduction of systolic BP of at least 20 mm Hg or diastolic BP of at least 10 mm Hg within 3 minutes of standing [20]. The diagnosis of OH was based solely on the baseline BP measurements.

By contrast, a diagnosis of hypertension was based on the medical history and the medical records only, and not on the baseline BP measurements.

The assessments took place during normal office hours (i.e. 8 a.m. to 4 p.m.).

### APOE

Apolipoprotein E (APOE) genotypes were determined in a subgroup. First, genomic DNA was extracted from 200 µl EDTA-blood using the QIAamp 96 DNA Blood Kit (Qiagen, Hilden, Germany). For detection of the APOE ε2, ε3 and ε4 genotypes, which are determined by the combination of two SNP’s (rs7412 and rs429358), we employed the LightCycler *APOE* Mutation Detection Kit (Roche Diagnostics, Mannheim, Germany), using the assay according to the instructions of the manufacturer.

### Assessment of Physical Comorbidity

We employed the “Cumulative Illness Rating Scale” (CIRS) for assessment of physical comorbidity. This instrument measures the chronic medical illness burden, while also taking into account the severity of chronic diseases. Scoring was done by an experienced geriatrician, in accordance with guidelines [35].

### MRI

Patients were scanned at three different sites; Stavanger University Hospital, Haugesund Hospital, and Haraldsplass Deaconess Hospital (Bergen). 1.5 T scanners were used in all three centres (Philips Intera in Stavanger and Haugesund, and in Bergen a 1.5T GE Signa Excite scanner). In each centre, MRI was done on the same scanner during the entire study period, and a common study imaging protocol was used. For technical details, see Soennesyn et al. [9]. A phantom study, using the same three scanners, of three human volunteers was done for the DemWest study and has recently been published [36]. This was done to assess the variability between scanners and also to assess intra-scanner variability. Cronbach’s alpha between the three MRI scanners, as well as between two points in time, all exceeded 0.95, indicating excellent reliabilities.

The MRI scans were performed within a median interval of 2 months (interquartile range 1–4 months) from the baseline clinical examination.

#### Volumetric assessment of WMH

Image analysis was performed according to a method developed and previously published by Firbank et al. [4] and modified as previously described [9]. Briefly, this method requires sets of 3DT1 weighted scans and FLAIR images from each patient. Non-brain regions were removed from the T1 image, and the WMH were segmented on a slice-by-slice basis from the FLAIR image, using a threshold determined from the histogram of pixel intensities for each image slice. An MNI atlas image registered to the FLAIR image was used to calculate the WMH volumes in different regions of the brain.

Because of the variability in image quality from the different centres participating in this study, we found it difficult to empirically choose a single threshold level that gave us a perfect segmentation result in each subject. Therefore, a threshold level of 1.2 was chosen, by which the lesion load was overestimated. Later, manual correction was performed by removing excess pixels using FSLView (http://www.fmrib.ox.ac.uk/fsl/index.html).

A specialist in internal medicine and geriatrics (HS) performed the manual editing, blind to clinical data, after training by a consultant neuroradiologist (MKB). They both edited the same 10 datasets twice; once in the beginning, to secure good inter-rater reliability, and a second time at the end of the editing process, to secure that similar reliability still persisted and to evaluate intra-rater reliability. The intraclass correlation coefficient (ICC) was calculated to be 0.998 for inter-rater reliability and 0.964 for intra-rater reliability. The manually edited scans were then used in the further analyses of volumes of total and regional WMH. In order to compensate for interindividual differences in total brain volumes, we calculated the ratios of volumes of WMH to total brain volumes, using these in the statistical analyses. In the present study, we used only the ratios of total WMH volumes, which have been shown to be highly correlated with regional WMH volumes [37].

#### Visual assessment of WMH

MRI’s were also rated visually, using the Scheltens scale [38], by an experienced rater (OJG), blind to clinical data. According to the Scheltens scale, white matter changes (WMC) are subdivided into periventricular WMC and deep WMC, and deep WMC are further subdivided into deep WMH (DWMH), basal ganglia WMH (BGH) and infratentorial hyperintensities (IT) [39]. In the statistical analyses, we used only the DWMH scores, because these have been associated with (orthostatic) BP drop in previous studies [15], [17]. Inter-rater reliability with another experienced rater (MKB) was evaluated, based on 12 scans, finding an ICC of 0.923.

### Statistical Analyses

A total of 82 patients had MRI scans that could be analysed volumetrically (volumetry group), and 139 had scans that could be rated semi-quantitatively (the semi-quantitative group) according to the Scheltens scale. The scans of 61 patients were analysed with both methods, yielding a correlation coefficient (Spearman’s rho) of 0.791 (p<0.001) between the scores of the two methods. Mann-Whitney U-test, Chi-square, Spearman rank order or Fisher’s exact test were used as appropriate. None of the continuous variables had a normal distribution, according to the Kolmogorov-Smirnov test.

Potential predictor variables having p-values <0.25 in bivariate logistic regression analyses were included in stepwise multiple logistic regression analyses, with the response variable defined as being in the highest quartile of total WMH volume ratios or Scheltens deep WMH (DWMH) scores vs. the lowest quartile, respectively.

P-values <0.05 (two-tailed) were considered statistically significant.

All statistical tests were performed using PASW Statistics 18, release 18.0.1.

## Results

When comparing the baseline characteristics of patients undergoing WMH volume analysis with those who were not included in the study, the only significant difference was a higher proportion with Alzheimer’s disease among the participants (volumetry group: Pearson Chi square 14.558, df 1, p<0.001, semi-quantitative group: Pearson Chi square 8.162, df 1, p = 0.006 (Table 1)).

**Table 1:** Demographic and clinical characteristics.

	Total sample n = 246	Volumetry group (n = 82), vs. rest of sample	Semi-quantitative group (n = 139), vs. rest of sample	Missing data (out of n = 246)
Age, median (IQR)	76.9 (71–81)	76.1 (70–81), p = 0.502	76.5 (71–81), p = 0.562	0
Women, n (%)	139 (57)	51 (62), p = 0.256	84 (60), p = 0.198	0
MMSE, median (IQR)	24 (22–26)	24 (22.5–26), p = 0.217	23.3 (22–25), p = 0.377	5
Coronary heart disease, n (%)	49 (21)	15 (19), p = 0.704	27 (21), p = 0.949	16
Hypertension (history of), n (%)	109 (46)	30 (38), p = 0.115	63 (47), p = 0.831	11
Diabetes mellitus, n (%)	21 (9)	9 (11), p = 0.495	10 (7), p = 0.455	12
APOEε4≥1 allele, fractions (%)	93/153 (61)	31/53 (59), p = 0.803	61/98 (62), p = 0.748	93
Previous stroke, n (%)	33 (14)	8 (10), p = 0.302	18 (14), p = 0.960	14
Smoker (former/pres.), n (%)	111 (48)	37 (47), p = 0.862	62 (48), p = 0.949	16
Heart failure, n (%)	12 (5)	2 (3), p = 0.227	5 (4), p = 0.416	22
Orthostatic hypotension (present), n (%)	90 (46)	35 (47), p = 0.945	49 (44), p = 0.727	49
CIRS score, median (IQR)	6 (4–8)	6 (4–7), p = 0.402	6 (4–7), p = 0.780	10
No. of drugs, median (IQR)	4 (2–6)	4 (2–5), p = 0.159	4 (2–5), p = 0.466	11
Blood pressure lowering medication*, n (%)	141 (60)	41 (53), p = 0.167	77 (57), p = 0.361	9
Dementia categories		**p = 0.000**	**p = 0.002**	0
Alzheimer’s disease, n (%)	138 (56)	60 (73)	89 (64)	
DLB/PDD, n (%)	89 (36)	16 (20)	38 (27)	
Vascular dementia, n (%)	11 (4)	2 (2)	5 (4)	
FTD/alcoholic dem., n (%)	8 (3)	4 (5)	7 (5)	

IQR = interquartile range; MMSE = Mini-Mental State Examination, normal range 24–30; AD = Alzheimer’s Disease; DLB = Dementia with Lewy Bodies; PDD = Parkinson’s Disease Dementia; VaD = vascular dementia; FTD = Frontotemporal Dementia; CIRS = Cumulative Illness Rating Scale, range 0 (no impairment)-52 (extremely severe impairment); APOE = Apolipoprotein E.

* antianginals, antihypertensives, tricyclic antidepressants, paroxetine,MAO inhibitors, dopamine agonists, diazepam, dipyridamole, phenothiazines, clozapine, quetiapine, haloperidol.

Significant results are shown in **bold** typeface.

In the volumetry group, the only significant difference with respect to relevant clinical characteristics between patients in the highest and lowest WMH quartiles was a lower proportion in the former group with at least one APOEε4 allele (Table 2).

**Table 2:** Demographic and clinical characteristics, lowest vs. highest WMH quartile.

	Volumetry group	Semi-quantitative group
OH (fractions)	10/17, 10/19 p = 0.970	12/25, 14/28 p = 1.000
Systolic BP drop (median)*	10, 10 p = 0.949	10, 17.5 p = 0.492
Diastolic BP drop (median)*	0, 0 p = 0.308	3, 0 p = 0.158
Standing syst. BP≤110 (fractions)	1/17, 2/19 p = 1.000	4/26, 2/28 p = 0.413
Age (median)*	73, 79.5 p = 0.081	72, 78.4 **p = 0.002**
Women (fractions)	15/20, 13/20 p = 0.730	19/37, 17/31 p = 0.966
AD (fractions)	16/20, 14/20 p = 0.715	22/37, 22/31 p = 0.463
Hypertension (fractions)	6/18, 11/20 p = 0.310	10/36, 16/31 p = 0.081
Coronary heart disease (fractions)	1/19, 5/20 p = 0.182	6/35, 7/30 p = 0.756
Diabetes mellitus (fractions)	1/19, 1/20 p = 1.000	3/36, 2/31 p = 1.000
APOEε4≥1 allele (fractions)	11/12, 6/13 **p = 0.030**	17/36, 10/21 p = 0.353
Previous stroke (fractions)	2/20, 4/19 p = 0.407	2/34, 9/28 **p = 0.016**
Smoker (former or present)(fractions)	8/20, 11/20 p = 0.527	19/33, 16/30 p = 0.933
Heart failure (fractions)	0/20, 1/18 p = 0.474	2/34, 1/28 p = 1.000

WMH = white matter hyperintensities; OH = orthostatic hypotension; BP = blood pressure; AD = Alzheimer’s disease; APOE = apolipoprotein E.

* Mann-Whitney U-test, all other comparisons Chi-Square or Fisher’s Exact test.

Significant results are shown in **bold** typeface.

In the semi-quantitative group, patients in the highest DWMH score quartile were significantly older than those in the lowest quartile, and the proportion of patients with a previous stroke was significantly higher in the highest quartile. Otherwise, there were no significant differences between those belonging to the highest and lowest DWMH score quartiles.

We did not find any significant association between a history of hypertension and having OH at baseline (Pearson Chi Square 0.224, df 1, p = 0.636).

### Associations between WMH and OH

There was no significant correlation between WMH volume ratios and the systolic orthostatic BP drops (Spearman’s rho 0.022, p = 0.848), but a trend with diastolic orthostatic BP drops was demonstrated (Spearman’s rho −0.213, p = 0.066). Similarly, we found no significant correlations between DWMH scores and systolic or diastolic orthostatic BP drops (Spearman’s rho 0.037, p = 0.700 and Spearman’s rho −0.122, p = 0.202, respectively).

We performed bivariate logistic regression analyses with the variables in Table 2 as predictors, and being in the highest WMH quartile vs. the lowest quartile as response variable. In the volumetry group, age, hypertension, coronary heart disease and APOEε4 status had p-values <0.25. As to the semi-quantitative group, age, hypertension, APOEε4 status and previous stroke had p-values <0.25. None of the p-values for the BP variables approached this level, except diastolic BP drop vs. DWMH score (p = 0.297). The aforementioned variables having p-values <0.25 were entered into stepwise multiple logistic regression analyses.

In the final model, only APOEε4 status remained a significant predictor of the volumes of WMH (Table 3). The model performed well (Omnibus test of model coefficients p<0.05), and the model fit was good (Generalised linear models, Pearson Chi Square p = 0.179). Only age remained a significant predictor of DWMH scores (Table 4). The model performed well (Omnibus test of model coefficients p = 0.010), and the model fit was good (Hosmer and Lemeshow test p = 0.492).

**Table 3:** Multiple logistic regression analysis of the effect of APOEε4 status on likelihood of having WMH volume in highest vs. lowest quartile (final model).

	B	S.E.	p	Odds Ratio Exp. (B) (95% CI)
≥1 APOEε4 allele	−2.595	1.242	0.037	0.075 (0.007–0.851)

APOE = apolipoprotein E; WMH = white matter hyperintensities; CI = confidence interval.

**Table 4:** Multiple logistic regression analysis of the effect of age on likelihood of having Scheltens deep WMH score in highest vs. lowest quartile (final model).

	B	S.E.	p	Odds Ratio Exp. (B) (95% CI)
Age (years)	0.112	0.048	0.019	1.119 (1.018–1.230)

APOE = Apolipoprotein E; WMH = white matter hyperintensities; CI = confidence interval.

We also performed multiple logistic regression analyses (stepwise and forced entry) controlling for scanning site and including variables known from previous studies to be associated with WMH (age, hypertension, diabetes mellitus), in addition to OH or systolic or diastolic BP drops. In these analyses, both with respect to the volumetry group and the semi-quantitative group, only age remained a significant predictor of WMH load (data not shown). However, in some of the models the predictor “MRI centre” achieved borderline significance (p = 0.048–0.050).

When analysing the patients with DLB/PDD separately, we found no significant correlations between Scheltens DWMH scores and systolic or diastolic BP drops. Similarly, there were no significant differences between those in the highest and lowest Scheltens DWMH score quartiles with respect to the other variables in Table 2 (data not shown). In bivariate logistic regression analyses, diastolic BP drop, age and APOEε4 status achieved the lowest p-values (0.124, 0.117 and 0.094, respectively). Due to the rather small subsample, in combination with missing values for the relevant variables, it was not statistically feasible to perform multiple logistic regression analyses using these variables.

## Discussion

The main finding of our study is that in this sample of older people with mild dementia, WMH were not associated with OH or low standing systolic BP. Only APOEε4 status (volumetry) and age (volumetry and semi-quantitative analysis) were independently associated with WMH volumes.

Thus, our hypothesis that WMH in mild dementia are associated with OH was not supported. This finding is in contrast to some previous studies. However, some of these studies were performed in older people with major depression [16], [18], [19], whereas in our study only a minority (17%) had clinically significant depression (defined as a Montgomery Asberg Depression Rating Scale [40] score of at least 15). In the other studies [15], [17], a majority of the relevant subjects had DLB, known to have lower standing systolic BP values than AD [23], whereas in our study the majority had AD. Furthermore, in these two last studies blood pressures were measured partly or only during carotid sinus massage, as opposed to our study, in which the blood pressures were measured only in the supine (or sitting) position and during active standing. Subjects with OH according to these two different methods may not be comparable, e.g. concerning the pathophysiology of WMH.

In our study, the presence of at least one APOEε4 allele was associated with reduced odds of having high WMH volume, suggesting that other APOEε alleles (i.e. ε2 and/or ε3) may increase the odds of high WMH volume. This hypothesis is supported by at least two previous studies [41], [42]. Notably, none of these included subjects with dementia. Alternatively, patients possessing the ε4 allele may have more neurodegenerative changes and thus develop dementia with a lower WMH load. However, the majority of studies in this field have not demonstrated any association between APOEε4 status and WMH burden [43]–[48].

In contrast to some previous studies (e.g. [49]), we did not find any significant associations between hypertension and WMH. This could have several possible explanations, including different definitions of hypertension, different study designs, and differences regarding samples.

This being a multicentre study, it is possible that the measured or scored WMH values might vary systematically according to scanning site. The results of the phantom studies, as well as the results of the multivariate analyses including scanning site as a variable, do not support this hypothesis.

The strengths of our study include the use of both quantitative and semi-quantitative methods for evaluation of WMH severity. Furthermore, we had data on a number of potential causal or risk factors for WMH, enabling us to include these in the analyses.

Limitations include the cross-sectional design, the relatively small sample size, and orthostatic BP measurements in a number of cases obtained from the sitting, instead of the supine position. It has previously been demonstrated that sit-stand testing for OH has a very low diagnostic accuracy [50]. However, sit-stand measurement only has been used in recent, similar studies [51], [52]. In addition, no standing BP measurements were made after 3 minutes. According to a previous study [53], at least 20–30% of dementia patients have a delayed orthostatic response. Thus, our methodology would tend to underestimate the prevalence of OH, thereby possibly masking the potential association between OH and WMH. Furthermore, the consensus definition of OH, which was employed in the present study, does not in itself require the orthostatic BP to be measured on more than one occasion. This is a potential limitation, as this approach cannot distinguish those having only transient OH from those having more persistent or frequently recurring OH. The latter groups may have a higher risk of being afflicted with the potential adverse consequences of BP drops, such as syncope and cerebral hypoperfusion, and possibly also the development of WMH. Ideally, in order to identify individuals with more than transient OH, orthostatic blood pressures should have been measured repeatedly over a period of e.g. a few weeks. Moreover, if OH does play a role in the development of WMH in mild dementia, it probably exerts its effects over an extended period of time, also prior to the diagnosis of dementia. Exploring this clearly would require a longitudinal study. One final point is that due to missing data for some variables, a relatively low number of subjects could be included in the multiple logistic regression analyses, thus limiting the number of predictors that could be entered into these analyses, as well as their power.

Our results suggest that OH or low standing BP may not be associated with WMH in older people with mild dementia, at least not cross-sectionally. Instead, these changes may primarily be associated with neurodegenerative disease [14], ageing [54], hypertension and smoking [2], [11], genetics [55], or combinations of these factors. However, recent longitudinal studies indicate that an unfavourable vascular risk factor status from midlife and onwards may be of importance for the development of WMH in later life [10], [56], [57]. Thus, the best opportunities for potential prevention of these changes may lie in controlling established vascular risk factors, starting no later than in midlife.

### Conclusion

In a sample of older people with mild dementia, we found no cross-sectional association between OH and WMH load. Future studies should include larger samples, use a longitudinal design, and use more rigorous BP measurement protocols.
